# First Draft Genome Sequence from a Member of the Genus *Agrococcus*, Isolated from Modern Microbialites

**DOI:** 10.1128/genomeA.00391-13

**Published:** 2013-06-27

**Authors:** Richard Allen White, Christopher J. Grassa, Curtis A. Suttle

**Affiliations:** Department of Microbiology & Immunology, University of British Columbia, Vancouver, British Columbia, Canadaa; Department of Botany, University of British Columbia, Vancouver, British Columbia, Canadab; Department of Earth, Ocean & Atmospheric Science, University of British Columbia, Vancouver, British Columbia, Canadac; Canadian Institute for Advanced Researchd ‡

## Abstract

We report the first draft genome sequence from a member of the genus *Agrococcus*, isolated from cold thrombolytic microbialites within Pavilion Lake, British Columbia, Canada. The draft genome assembly for *Agrococcus pavilionensis* strain RW-1 has a size of 2,878,403 bp with a G+C content of 72.56%.

## GENOME ANNOUNCEMENT

Groth et al. (1) first described the genus *Agrococcus* based on two strains of *Agrococcus jenensis* isolated from soil and the surface of sandstone. The genus *Agrococcus* is classified within the family *Microbacteriaceae*, members of which exhibit the unusual feature of having diaminobutyric acid within the cell wall, which imparts a distinctive yellow color (1). The genus has eight described species that have been isolated from a wide range of environments, including air (2), a coal mine (3), cheese (4), cold desert soil (5), forest soil (6), a medieval wall painting (7), dried seaweed (8), and the phyllosphere of potato plants (9). The genetic richness and diversity of this genus is poorly known, with little genetic information available other than for the 16S rRNA genes.

The source of *Agrococcus pavilionensis* strain RW-1 is the oligotrophic (3.3 µg liter^-1^ total phosphorus) Pavilion Lake (50.86677°N, 121.74191°W), which lies in Marble Canyon near Lillooet, British Columbia, Canada. Pavilion Lake harbors a diverse array of modern microbialites, which are contemporary biogenically derived carbonate structures (10, 11). Pavilion Lake microbialites consist mainly of clotted and nonlayered thrombolitic structures (10) that occur in the permanently cold (4 to 8°C) water deeper than 5 m (11). *A. pavilionensis* was isolated from a cabbage-shaped thrombolite collected at a depth of 20 m. DNA was extracted using Qiagen QIAamp followed by Qiagen MinElute cleanup columns. The Illumina MiSeq library was constructed using the Lucigen NxSeq library prep kit without final PCR enrichment.

Whole-genome shotgun sequencing was completed using Illumina MiSeq in the 250-bp paired-read format. A partial flow cell obtained 2.89 million raw reads with 713,936,519 bp of raw sequence. Paired reads were error corrected and connected using AllPaths-LG (version 44837) (12). In the data set, 31mers were counted using Jellyfish (version 1.1.10) (13). Reads that contained 31mers with a multiplicity of >1,250 were partitioned for *de novo* assembly. The partitioned reads were assembled using Celera assembler 7.0 (14). The high-copy reads assembled as a single contig of 1,427 bp in length representing a high-copy plasmid. The remaining reads assembled as 50 contigs summing 2,878,403 bp (*N*_50_ length, 133,224; *N*_90_ length, 31,609; G+C content, 72.56%). The 16S rRNA gene sequence was confirmed by Sanger sequencing and was found to have 99.99% identity to the 16s rRNA gene predicted from the draft genome.

Annotation was conducted on the RAST server using the Glimmer 3 option (15) and predicted 2,506 protein-coding genes, including 48 noncoding RNA genes and 126 predicted SEED subsystem features. The potential to metabolize a wide range of carbon compounds is predicted from the genome, including d-ribose, fructose, lactate, glycerate, chitin, deoxyribose, and deoxynucleoside catabolism. Genes related to those encoding the phosphate (Pho) regulon for high-affinity uptake of phosphate and cold shock proteins were also found.

Further analysis of the genome, including functional and biochemical measurements, will be used to understand the possible roles of *A. pavilionensis* in the highly diverse microbial community contained within the Pavilion Lake microbialites. This is the first draft genome for the genus *Agrococcus*, which will provide a template for many further phylogenetic, comparative genomic, metagenomic, and functional studies of this widely distributed genus.

### Nucleotide sequence accession numbers.

This Whole-Genome Shotgun project has been deposited at DDBJ/EMBL/GenBank under the accession no. ASHR00000000. The version described in this paper is version ASHR01000000.
